# Potential Efficacy of Chitosan-Poly (Lactide-Co-Glycolide)-Encapsulated Trivalent Immersion Vaccine in Olive Flounder (*Paralichthys olivaceus*) Against Viral Hemorrhagic Septicemia Virus, *Streptococcus parauberis* Serotype I, and *Miamiensis avidus* (Scuticociliate)

**DOI:** 10.3389/fimmu.2021.761130

**Published:** 2021-12-02

**Authors:** Sajal Kole, Showkat Ahmad Dar, Su-Mi Shin, Hyeon-Jong Jeong, Sung-Ju Jung

**Affiliations:** Department of Aqualife Medicine, Chonnam National University, Yeosu, South Korea

**Keywords:** *Streptococcus parauberis*, *Miamiensis avidus*, VHSV, olive flounder, chitosan–PLGA

## Abstract

Olive flounder (*Paralichthys olivaceus*) is the most valuable aquaculture species in Korea, corresponding to ~60% of its total production. However, infectious diseases often break out among farmed flounders, causing high mortality and substantial economic losses. Although some deleterious pathogens, such as *Vibrio* spp. and *Streptococcus iniae*, have been eradicated or contained over the years through vaccination and proper health management, the current disease status of Korean flounder shows that the viral hemorrhagic septicemia virus (VHSV), *Streptococcus parauberis*, and *Miamiensis avidus* are causing serious disease problem in recent years. Furthermore, these three pathogens have differing optimal temperature and can attack young fingerlings and mature fish throughout the year-round culture cycle. In this context, we developed a chitosan-poly(lactide-co-glycolide) (PLGA)-encapsulated trivalent vaccine containing formalin-killed VHSV, *S. parauberis* serotype-I, and *M. avidus* and administered it to olive flounder fingerlings by immersion route using a prime-boost strategy. At 35 days post-initial vaccination, three separate challenge experiments were conducted *via* intraperitoneal injection with the three targeted pathogens at their respective optimal temperature. The relative percentages of survival were 66.63%, 53.3%, and 66.75% in the group immunized against VHSV, *S. parauberis* serotype-I, and *M. avidus*, respectively, compared to the non-vaccinated challenge (NVC) control group. The immunized fish also demonstrated significantly (*p* < 0.05) higher specific antibody titers in serum and higher transcript levels of Ig genes in the mucosal and systemic tissues than those of NVC control fish. Furthermore, the study showed significant (*p* < 0.05) upregulation of various immune genes in the vaccinated fish, suggesting induction of strong protective immune response, ultimately leading to improved survival against the three pathogens. Thus, the formulated mucosal vaccine can be an effective prophylactic measure against VHS, streptococcosis, and scuticociliatosis diseases in olive flounder.

## 1 Introduction

Disease prevention is the key to improving production and profitability in aquaculture worldwide. Vaccination is generally accepted as the most effective prophylactic measure for disease prevention in fish based on environmental, social, and economic grounds (1). However, with the increase in variation of pathogens affecting a single fish species during its year-round culture cycle, it is imperative to develop multivalent vaccines to protect the cultured stock in a cost-effective manner using a single-vaccination program. In Korea, olive flounder (*Paralichthys olivaceus*) is one such economically important aquaculture species, which contributes approximately 58.1% of Korea’s total aquaculture production (2), but it is affected by various pathogenic diseases throughout the culture cycle, causing huge economic losses every year. The four most serious diseases having the maximum detrimental impacts on the flounder industry over the last two decades are viral hemorrhagic septicemia (VHS) caused by viral hemorrhagic septicemia virus (VHSV), an enveloped (−) ssRNA virus belonging to the genus *Novirhabdovirus* of family Rhabdoviridae (3); streptococcosis caused by Gram-positive bacteria *Streptococcus iniae* and *Streptococcus parauberis* (4, 5); edwardsiellosis caused by Gram-negative bacteria *Edwardsiella tarda* (*E. piscicida*) (6); and scuticociliatosis caused by the voraciously histophagous ciliate *Miamiensis avidus* (syn. *Philasterides dicentrarchi*) of phylum Ciliophora, subclass Scuticociliatida (7, 8). Moreover, recent epidemiological studies demonstrated that *S. iniae* has disappeared from Korean flounder farms in recent years, but on the contrary, the prevalence of *S. parauberis* has increased multiple times with two distinct serotypes, I (contributing ~64%) and II (contributing ~36%) (4). Thus, this study initially aimed to develop a multivalent vaccine against VHSV, *S. parauberis* serotype I, *E. tarda*, and *M. avidus*, but due to the low efficacy (i.e., no protection) of the vaccine against *E. tarda*, we excluded the *E. tarda* antigen and re-formulated the vaccine against three pathogens.

Although the three pathogens are equally deleterious to olive flounder, their infection pathologies, temperature susceptibilities, and economic impacts differ. In brief, VHSV outbreaks occur during late winter and spring when the water temperature is approximately 8–15°C, causing dark coloration, ascites, hemorrhages on external body surfaces, congested liver, and swelling of the spleen and kidney (9, 10), ultimately resulting in 50%–70% mortality in all age groups of flounder in a very short time. In contrast, outbreaks of streptococcosis caused by *S. parauberis* serotype I take place throughout the year and display no pathological characteristics except for darkening of the skin, but they quickly lead to high mortality irrespective of flounder size (11–14). Moreover, scuticociliatosis disease, which also occurs year-round, causes severe hemorrhages and ulcers in the skin, skeletal muscles, fins, gills, and jaw, and the parasite frequently invades internal body parts, such as the brain, ascites, and spinal cord, thus causing mortality in young fingerlings and resulting in a high (46%–57%) cumulative loss to the flounder industry (8, 15).

Therefore, to overcome these diseases and sustain flounder production, development of an effective vaccine containing trivalent antigens is urgently needed. Furthermore, it is also important that the developed vaccine uses non-stressful delivery mechanism so that it can be administered to fish of all sizes, particularly young fingerlings, where high mortality is frequently observed due to VHSV and scuticociliate infection. Previously, we developed encapsulated VHSV vaccines using chitosan and (poly)lactide-co-glycolide (PLGA) nanoparticles, which gave moderate-to-high protective efficacy post-immersion vaccination in olive flounder (16, 17). However, in recent studies, the use of a chitosan–PLGA complex as an encapsulation material for fish mucosal vaccination has been gaining popularity as it aids in exploiting the mucoadhesive property of the nanoparticle complex, in turn facilitating efficient administration of the vaccine *via* skin and gill surfaces with minimum antigen leakage (18–21). Taking a cue from these studies, the present study was conducted to develop a chitosan–PLGA-encapsulated trivalent vaccine complex containing inactivated VHSV, *S. parauberis* serotype I, and *M. avidus*, which was administered to olive flounder fingerlings *via* immersion route in a prime-boost manner to evaluate its ability to deliver antigens to the host immune cells and induce protective immunity against the three pathogens in olive flounder.

## 2 Materials and Methods

### 2.1 Experimental Animals

Olive flounder (*P. olivaceus*) fingerlings (10.5 ± 1.5 g) obtained from a local farm were treated with 50 ppm formalin and acclimatized in seven 250-L fiber-reinforced (FRP) tanks in our indoor rearing re-circulation facility provided with ultraviolet (UV)-treated aerated seawater, maintained at 19–20°C water temperature and pH 8.0–8.2. Fish were fed twice per day with a standard pellet diet at 3% of their body weight for 2 weeks prior to the vaccination trial. Pathogen-free status of the procured fish was confirmed by screening for viruses, bacteria, and parasites from 10 randomly selected fish as described in our previous study (16).

### 2.2 Antigen Preparation

#### 2.2.1 VHSV Antigen

VHSV (F1Wa05 strain) was propagated in fathead minnow (FHM) epithelial cell line in 75 cm^2^ tissue culture flasks at 15 ± 0.5°C. The culture was maintained with Dulbecco’s modified Eagle medium (DMEM; Gibco, Invitrogen, USA) supplemented with 10% (v/v) fetal bovine serum (FBS; Gibco), 100 IU/ml penicillin G, and 100 µg/ml streptomycin (Gibco). After the development of complete cytopathic effect (CPE), the cell culture supernatant was centrifuged at 3,500×*g* for 30 min at 4°C, and aliquots were stored at −80°C until use. The harvested VHSV with a virus titer of 10^8.8^ TCID_50_/ml was precipitated using polyethylene glycol (PEG) and NaCl (22). Briefly, 195 ml of VHSV (dose optimized at 7.5 × 10^7.8^ virus/fish/immunization dose) was mixed with 7% (w/v) PEG-6000 and 2.3% NaCl and gently stirred overnight in a magnetic stirrer at 4°C. After PEG precipitation, the virus mixture was centrifuged at 4,000×*g* for 1.5 h at 4°C. The supernatant was discarded carefully, and the viral pellet was dissolved in 8 ml of phosphate-buffered saline (PBS). To remove PEG, the dissolved pellet solution was then subjected to dialysis in PBS overnight at 4°C using Slide-A-Lyzer Dialysis Cassettes (Thermo Fisher Scientific, USA). The dialyzed virus was then inactivated by stirring with 0.3% formalin for 24 h at 4°C on a magnetic stirrer. Aliquots of 4 ml (4.875 × 10^9.8^ virus/ml) of inactivated virus (IV) antigen were stored at 4°C until use.

#### 2.2.2 *S. parauberis* Serotype I Antigen

*S. parauberis* serotype I (*S. parauberis* type I, SP1DS strain), isolated in our laboratory from infected olive flounder collected from Jeju island in 2013, was propagated in 1-L brain–heart infusion broth containing 1% NaCl at 25°C in a shaking incubator for 20 h. After the optical density at 540 nm (OD_540_) reached 1.3, the bacteria were harvested by centrifugation at 3,800×*g* for 1 h at 4°C. The bacterial pellet was washed with PBS twice by centrifugation at 3,800×*g* for 30 min at 4°C and subsequently dissolved in 100 ml of PBS. The bacteria were then inactivated by stirring with 0.5% formalin for 96 h at 4°C on a magnetic stirrer followed by adding 1 ml of sodium metabisulfite (1.5% SMS) and stirring for another 24 h. The inactivated bacterial cell pellet was collected by centrifugation at 3,800×*g* for 2 h at 4°C. The pellet was resuspended in 30 ml of PBS, and an aliquot of 2 ml (6.5 × 10^10^ CFU/ml; dose optimized at 5 × 10^8^ CFU/fish/immunization dose) was stored at 4°C until use.

#### 2.2.3 *M. avidus* Antigen

*M. avidus* (YS2 strain), isolated from the brain of an infected olive flounder (23), was maintained (sub-cultured at 45-day intervals) in the CHSE-214 cell line in a 75 cm^2^ tissue culture flask (Nunc, Denmark) at 10°C, where CHSE-214 cells served as food for the scuticociliate. The culture media used for maintenance consisted of DMEM (Gibco), 10% (v/v) FBS (Gibco), 50 IU/ml penicillin G, and 50 µg/ml streptomycin (Gibco). To increase ciliate production prior to the experiment, the incubation temperature was raised to 15°C along with subsequent sub-culturing at 5- to 7-day intervals. The scuticociliates propagated by feeding on CHSE-214 cells were harvested for vaccine preparation, counted using a hemocytometer, and inactivated by stirring with 0.05% formalin for 1 h at 4°C on a magnetic stirrer. An aliquot of 2.5 ml of inactivated scuticociliate containing 2.6 × 10^7^ cells (1.04 × 10^7^ cells/ml; dose optimized at 1 × 10^5^ cells/fish/immunization dose) was stored at 4°C until use.

### 2.3 Encapsulation of Antigens in Chitosan-Coated PLGA

Aliquots of inactivated VHSV (4 ml), inactivated *S. parauberis* type I (2 ml), and inactivated scuticociliate (2.5 ml) were mixed to form the water phase (W_1_) for the encapsulated vaccine. For the organic phase (O) (5% w/v), 1.5 g of PLGA (L:G = 50:50, M_W_ 30–60 kDa; Sigma-Aldrich, USA) was dissolved in 30 ml of dichloromethane (Sigma-Aldrich). Encapsulation was carried out as described by Charlie-Silva et al. (19) with some modification. Briefly, the water phase (W_1_) was homogenized with the organic phase (O) using a mechanical homogenizer (POLYTRON^®^ PT 1200E, Thomas Scientific, USA) at 2,500 rpm for 10 min to obtain the water-in-oil primary emulsion (W_1_/O). The resultant primary emulsion (W_1_/O) was further emulsified in 100 ml of aqueous polyvinyl alcohol (PVA) solution (5% w/v) to form a water-in-oil-in-water (W_1_/O/W_2_) emulsion. Nanospheres were prepared by homogenization of the W_1_/O/W_2_ emulsion for 10 min. After homogenization, the emulsion was kept overnight on a magnetic stirrer at 25°C to allow evaporation of the organic solvent. Then, a solution of low-molecular-weight chitosan (50–190 KDa; Sigma-Aldrich) at 5 mg/ml was added to the nanosphere suspensions with magnetic stirring for 1 h for coating. The chitosan-coated PLGA-encapsulated trivalent vaccine complex (Chi/PNPs-IV+Sc+Sp) was recovered by centrifugation at 5,000×*g* for 30 min at 4°C and washed three times (centrifugation at 5,000×*g* for 10 min at 4°C) with distilled water. The final product was stored at 4°C.

### 2.4 Vaccine Preparation

For primary and booster immunization *via* immersion, the vaccine complex (Chi/PNPs-IV+Sc+Sp) was suspended in 30 ml of distilled water prior to vaccination. Thus, the approximate antigen concentrations in 1 ml of the vaccine correspond to the following: VHSV: 3.25 × 10^8.8^ virus/ml; *S. parauberis* type I: 2.16 × 10^9^ CFU/ml; *M. avidus*: 4.33 × 10^5^ ciliates/ml.

### 2.5 Experimental Design for Immunization Trial

Olive flounder fingerlings (*n* = 260) (11.2 ± 0.5 g) were randomly distributed into two experimental groups with 130 fish per group and reared in 500-L FRP recirculating tanks supplied with UV-treated seawater maintained at 20°C. The groups were designated as Immersion and NVC control (non-vaccinated challenged) groups. For primary immunization, Immersion group fish were distributed in two plastic aquaria (*n* = 65 fish/aquarium), each containing 2 L of seawater with 15 ml of Chi/PNPs-IV+Sc+Sp vaccine solution (containing 4.875 × 10^9.8^ virus, 3.24 × 10^10^ CFU, and 6.495 × 10^6^ ciliate antigens), immersed for 2 h with vigorous aeration, and transferred back to the original tank after immersion. Fifteen days post-initial immunization (300 ⁰ days), a booster dose was administered using the same method. The NVC control group remained untreated.

### 2.6 Sampling

Three fish per time point from the Immersion and NVC control groups were randomly selected for sampling at 48 h post-initial vaccination (hpiv), 48 h post-booster vaccination (hpbv), and before challenge (35 days post-initial vaccination, dpiv). Blood serum, anterior kidney, spleen, gill, and skin (portion from caudal peduncle site) tissue samples were collected from both groups at each time point for further analysis of immune parameters.

### 2.7 Challenge Study

The challenge study was conducted separately for each of the three pathogens at their respective susceptible temperature conditions: 15°C for VHSV, 26°C for *S. parauberis* type I, and 20°C for scuticociliate. For each pathogen, 40 fish (17.0 ± 0.7 g) from the two experimental groups at 35 dpiv were transferred to the respective challenge facility in three plastic aquaria per group (two aquaria with 15 fish each for mortality study and one aquarium with 10 fish for post-challenge sampling) containing 25 L of UV-treated seawater. For the three experiments, fish from both the Immersion and NVC control groups were intraperitoneally injected with the respective pathogens homologous to the vaccine strain, with challenge doses as follows: VHSV, 100 µl of VHSV (10^5.8^ TCID_50_virus/fish); *S. parauberis* type I, 100 µl of *S. parauberis* type I (1.6 × 10^9^ CFU/fish); and *M. avidus*, 100 µl of scuticociliate (5 × 10^4^ cells/fish). Mortality patterns and clinical signs of VHS and streptococcosis were observed in each group daily for 20 days post-infection, whereas for scuticocilliosis, observation was performed for 27 days post-infection. The relative percentage of survival (RPS) was calculated by the following formula (24): RPS = [1 – (%Mortality in vaccinated group/%Mortality in control group)] × 100. From the remaining aquaria (apart from RPS analysis) in both groups of each challenge experiment, kidney and spleen tissue samples from three fish per time point were randomly sampled at 24, 48, and 96 h post-challenge (hpc), whereas blood serum was collected at 48 hpc.

### 2.8 Competitive ELISA for Specific Antibody Quantification in Experimental Fish

Specific antibody (against VHSV, *S. parauberis* type I, and *M. avidus*) quantification in the fish sera was performed using competitive enzyme-linked immunosorbent assays (c-ELISA), as described in our previous protocol (16) with required modifications. For VHSV c-ELISA, VHSV (10^8^ TCID_50_/ml) diluted in coating buffer (carbonate–bicarbonate buffer, pH 9.6) was used as the antigen. Serum samples (diluted at 1:40 in 1% bovine serum albumin [BSA] in PBS-T) were used as test samples; commercially available mouse monoclonal antibody (MAb) against VHSV glycoprotein (G; Enbiogene, Korea; 1:100 dilution in 1% BSA in PBS-T) was used as the competitive antibody. For *S. parauberis* type I c-ELISA, harvested bacteria diluted in PBS (adjusted to OD_540_ = 1) were used as the antigen. Serum samples (diluted at 1:40 in 1% BSA in PBS-T) were used as test samples; commercially available rabbit polyclonal antibody (PAb) against *S. parauberis* type I (Enbiogene) (1:200 dilution in 1% BSA in PBS-T) was used as the competitive antibody. For *M. avidus* c-ELISA, *M. avidus* (4.4 × 10^4^ cells/ml) diluted in coating buffer (carbonate–bicarbonate buffer, pH 9.6) was used as the antigen. Serum samples (diluted at 1:40 in 1% BSA in PBS-T) were used as test samples; diluted rabbit PAb (1:200 in 1% BSA in PBS-T) against *M. avidus* YS2 strain (previously developed in our laboratory) was used as the competitive antibody. Following ELISA, the OD was recorded at 492 nm using a VERSA max microplate reader (Perkin Elmer, USA). The results were expressed as percentage inhibition (PI), derived by the following formula: PI = 100 − (mean OD_492_ of test sample × 100)/(mean OD_492_ of MAb/PAb). The three c-ELISA procedures are described in detail in **Supplementary File 1**.

### 2.9 Immune Gene Expression

#### 2.9.1 RNA Isolation and cDNA Synthesis

Total RNA was extracted from the collected anterior kidney, spleen, gill, and skin samples from fish of two experimental groups (at 48 hpiv, 48 hpbv, and pre-challenge) as well as from anterior kidney and spleen samples of fish of both experimental groups per pathogen challenge at 24, 48, and 96 hpc using RNAiso Plus (Takara Bio Inc., Japan) according to the manufacturer’s protocols and quantified using a NanoDrop™ 1000 spectrophotometer (Thermo Fisher Scientific). The residual genomic DNA was removed using RNase-free DNase I (Takara Bio Inc.). Total RNA (1 µg) was reverse transcribed into first-strand cDNA using a ReverTra Ace^®^ qPCR RT Kit (Toyobo, Japan) with oligo-dT primer and ReverTra Ace reverse transcriptase in 10 µl reaction volume according to the manufacturer’s protocol. The resulting cDNA was stored at −20°C.

#### 2.9.2 Quantitative Expression Analysis of Immune Genes in Experimental Samples

Gene-specific primers for immune-related genes were designed using Primer3Plus based on available sequences from NCBI database and are listed in **Supplementary File 1**. To analyze gene expression, real-time PCR was performed in an Exicycler™ 96 Real-Time Quantitative Thermal Block (Bioneer, Korea) using SYBR Green AccuPower^®^ PCR PreMix (Bioneer). Relative quantification of immune gene expression was estimated using the 2^−ΔΔCt^ method (25). Real-time PCR is described in detail in **Supplementary File 1**.

### 2.10 Statistical Analysis

To analyze survival in the immunization experiment, Kaplan–Meier curve analysis and log rank test were carried out using GraphPad Prism5 Software, and survival curves were constructed in Microsoft Excel. The data generated for pathogen-specific antibody titers in serum and for gene expression in different tissue samples collected from the experiment groups at different time points were statistically analyzed using statistical package SPSS version 22 (SPSS Inc., USA). Each dataset was subjected to two-way analysis of variance (ANOVA) to determine the statistical significance within the group (time-wise) and between the groups (treatment-wise) as well as to evaluate the interaction effect. *Post-hoc* analysis followed by Duncan’s multiple range tests and an unpaired *t*-test were used to determine the significant differences in antibody titers and gene expression levels at different time points within and between the experimental fish groups. Statistical significance was set at *p* < 0.05. The results were expressed as mean ± standard error.

## 3 Results

### 3.1 Vaccine Efficacy and RPS

**Figure 1:**
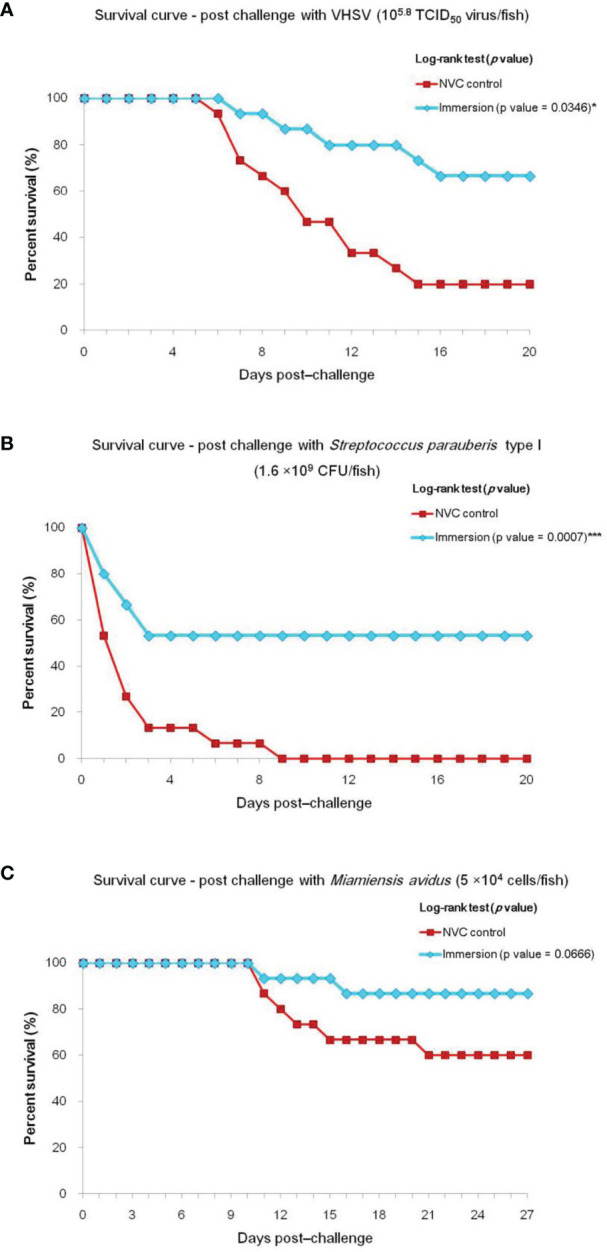
Survival curve of experimental trials showing cumulative mortality and relative percentage survival (RPS) of Immersion and NVC control groups of olive flounder (*n* = 30, two tanks with 15 fish/tank per group) challenged with virulent **(A)** VHSV (10^5.8^ TCID_50_ virus/fish); **(B)** *Streptococcus parauberis* type I (1.6 × 10^9^ CFU/fish); and **(C)** *Miamiensis avidus* (5 × 10^4^ cells/fish) at 35 dpiv. Significant differences (*) by log-rank test were noted between the NVC control group and the Immersion group in the challenge trials with VHSV and *S. parauberis* type I, with *p*-values of 0.0346 and 0.0007, respectively, whereas no significant difference (*p*-value = 0.0666) was observed in the *M. avidus* challenge trial. *** significance level of p < 0.001 for log-rank test.

To determine the efficacy of the chitosan–PLGA-encapsulated trivalent vaccine, olive flounders were immunized and then separately challenged with VHSV, *S. parauberis* type I, and *M. avidus*. In the VHSV challenge experiment, majority of the infected fish in the NVC control group showed visible ascites at 3–5 days post-challenge (dpc) with the first mortality occurring at 6 dpc, whereas in the immunized groups, 4–5 fish out of 30 fish showed apparent bulging of the abdominal cavity relatively later at 7–8 dpc, and the remaining fish showed no remarkable macroscopic signs. The vaccine efficacy study revealed 66.63% RPS for the immunized group compared with 80% cumulative mortality in the NVC control group (**Figure 1A**). In the *S. parauberis* type I challenge study, the maximum percentage of total mortality in both experimental groups occurred during 1–3 dpc, with 100% and 46.7% cumulative mortality in the NVC control and Immersion groups, respectively, and 53.3% RPS in the Immersion group (**Figure 1B**). Meanwhile, for the *M. avidus* challenge study, 26 of 30 fish in the vaccinated group were devoid of any pathological signs and remained stable throughout the 27 days post-challenge, with 66.75% RPS with respect to 40% cumulative mortality in the NVC control group (**Figure 1C**).

### 3.2 Specific Antibody Response

**Figure 2:**
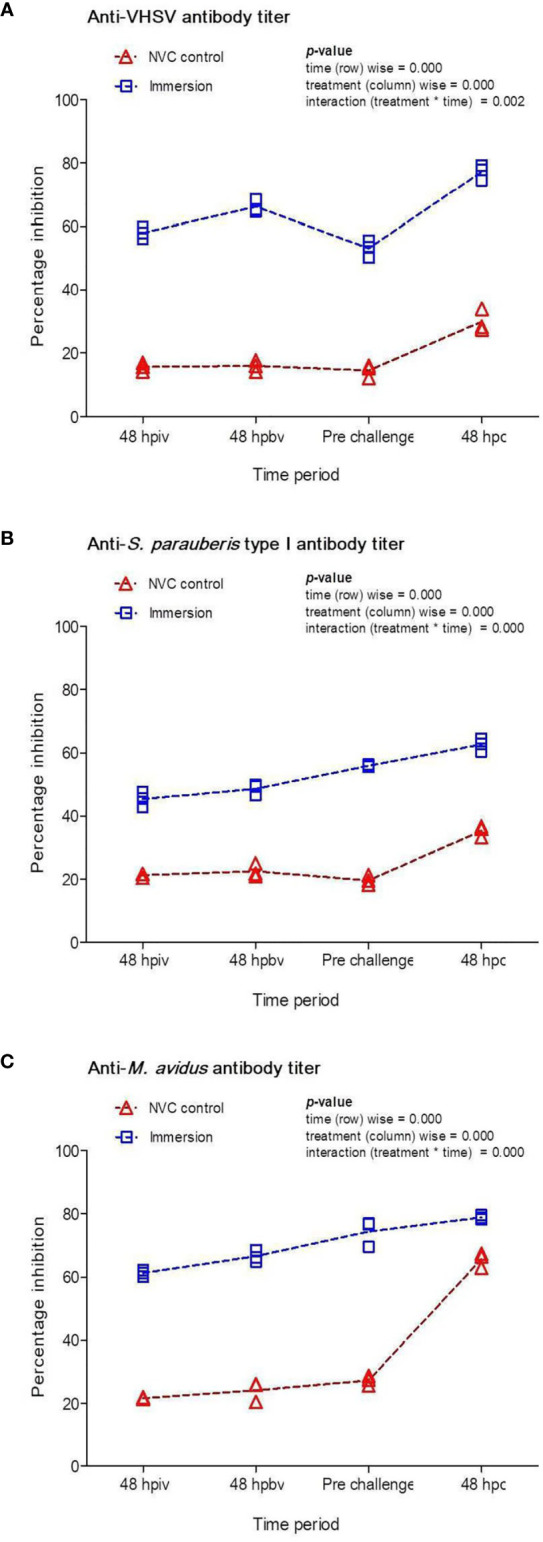
Percentage inhibitions (PI) of specific antibodies against **(A)** VHSV, **(B)** *S. parauberis* type I, and **(C)** *M. avidus* in the fish sera of Immersion and NVC control groups were determined using competitive ELISA with MAb against VHSV-G protein and PAbs against *S. parauberis* type I and *M. avidus* YS2 and plotted at different time intervals post-immunization with Chi/PNPs-IV+Sc+Sp vaccine complex as well as at 48 hpc with the three pathogens. The PI activities (*n* = 3) against respective pathogens in the serum were individually plotted with lines adjoining the respective mean ± standard error as a function of time. Two-way ANOVA was performed with the dataset, and the *p*-values within the group (time-wise), between groups (treatment-wise), and for the interaction effect were evaluated to determine statistical significance (*p* < 0.05).

To quantify the antigen-specific antibody titers, sera were obtained from the vaccinated and NVC control groups at 48 h post-initial and booster vaccination, pre-challenge (at 35 dpiv), and at 48 hpc. During the vaccination period, the NVC control group showed a relatively low anti-VHSV (**Figure 2A**) antibody titer (PI: 12%–14%) compared to both anti-*S. parauberis* type I (**Figure 2B**) and anti-*M. avidus* (**Figure 2C**) antibody titers (PI: 18%–24%). However, at 48 hpc, the scuticociliate-challenged control fish displayed a sharp and significant (*p* < 0.05) rise in antibody titer (PI: ~65%) compared to the modest (PI: ~30%) increase (*p* > 0.05) in the VHSV- and *S. parauberis* type I-challenged fish. In contrast, the immunized fish demonstrated a steady and significant (*p* < 0.05) increase in antibody titers post-immunization and post-challenge, in which the anti-VHSV (**Figure 2A**) and anti-*M. avidus* (**Figure 2C**) titers increased to ~60% PI after primary immunization and reached up to 80% PI at 48 hpc, whereas the anti-*S. parauberis* type I (**Figure 2B**) antibody titer recorded >40% PI post-immunization period and peaked at ~60% PI post-challenge.

### 3.3 Expression Kinetics of Immune-Related Genes Post-Immersion Vaccination

**Figure 3:**
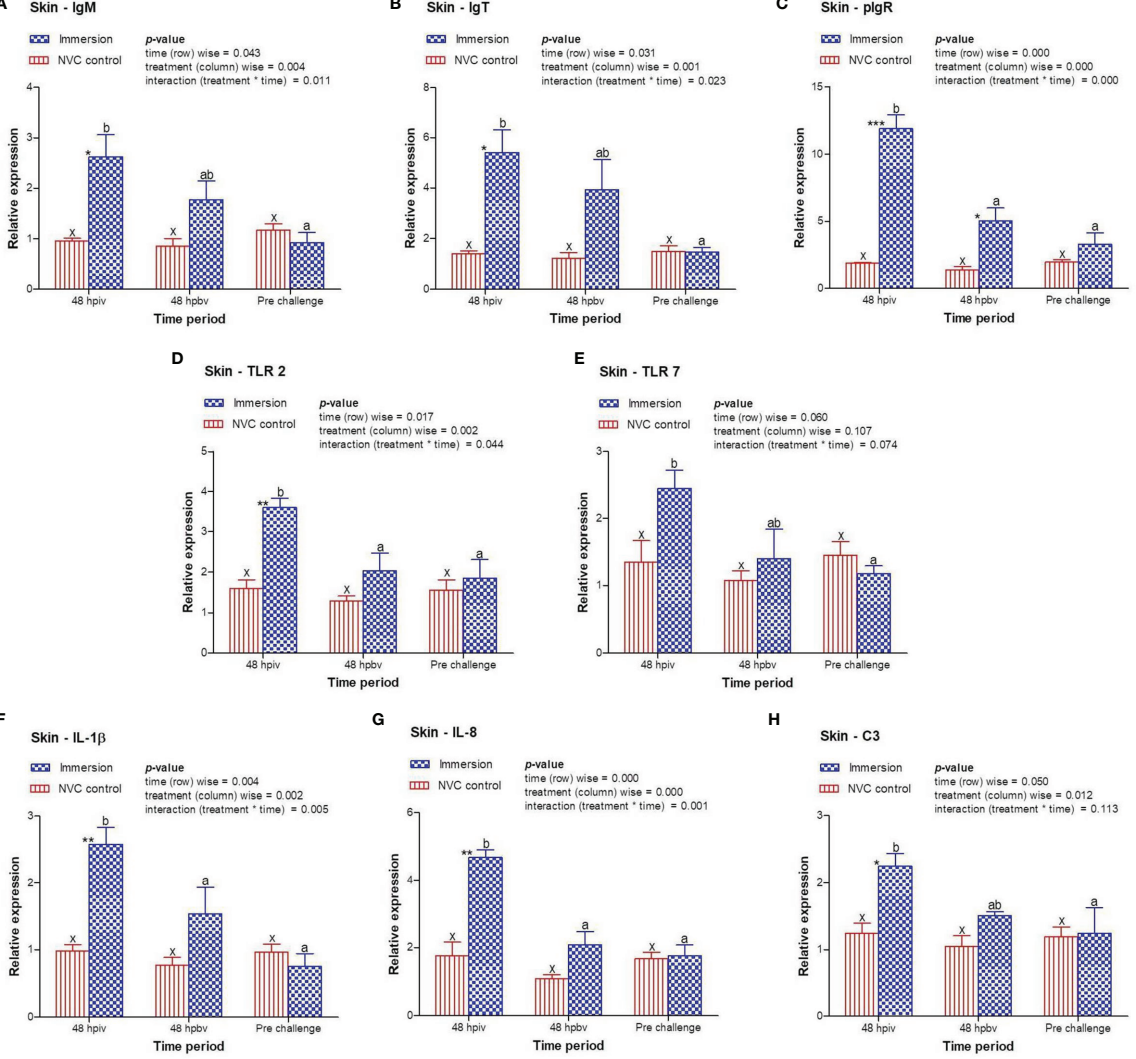
Relative expression of immune genes in skin tissue of olive flounder in the immersion vaccinated and NVC control groups. Expression levels of each gene were compared between the experimental groups relative to the naive control. The mean (*n* = 3) relative expression levels of IgM **(A)**, IgT **(B)**, pIgR **(C)**, TLR 2 **(D)**, TLR 7 **(E)**, IL-1β **(F)**, IL-8 **(G)**, and C3 **(H)** were plotted with standard error at different time intervals post-immunization with the chitosan–PLGA-encapsulated trivalent vaccine. Two-way ANOVA was performed with each dataset. The *p*-values within the group (time-wise), between groups (treatment-wise), and for the interaction effect were evaluated to determine statistical significance (*p* < 0.05). Duncan’s multiple range test [homogenous subsets indicated by lowercase (x-z) for NVC control and (a-c) for Immersion group] within the groups (time-wise) and an unpaired *t*-test (indicated by asterisks * for significance level) between the groups at different time points were also performed to analyze the statistical differences. * denotes significance level of t-test between the 2 groups viz., *, **, *** are for p < 0.05 , 0.01 and 0.001, respectively.

**Figure 4:**
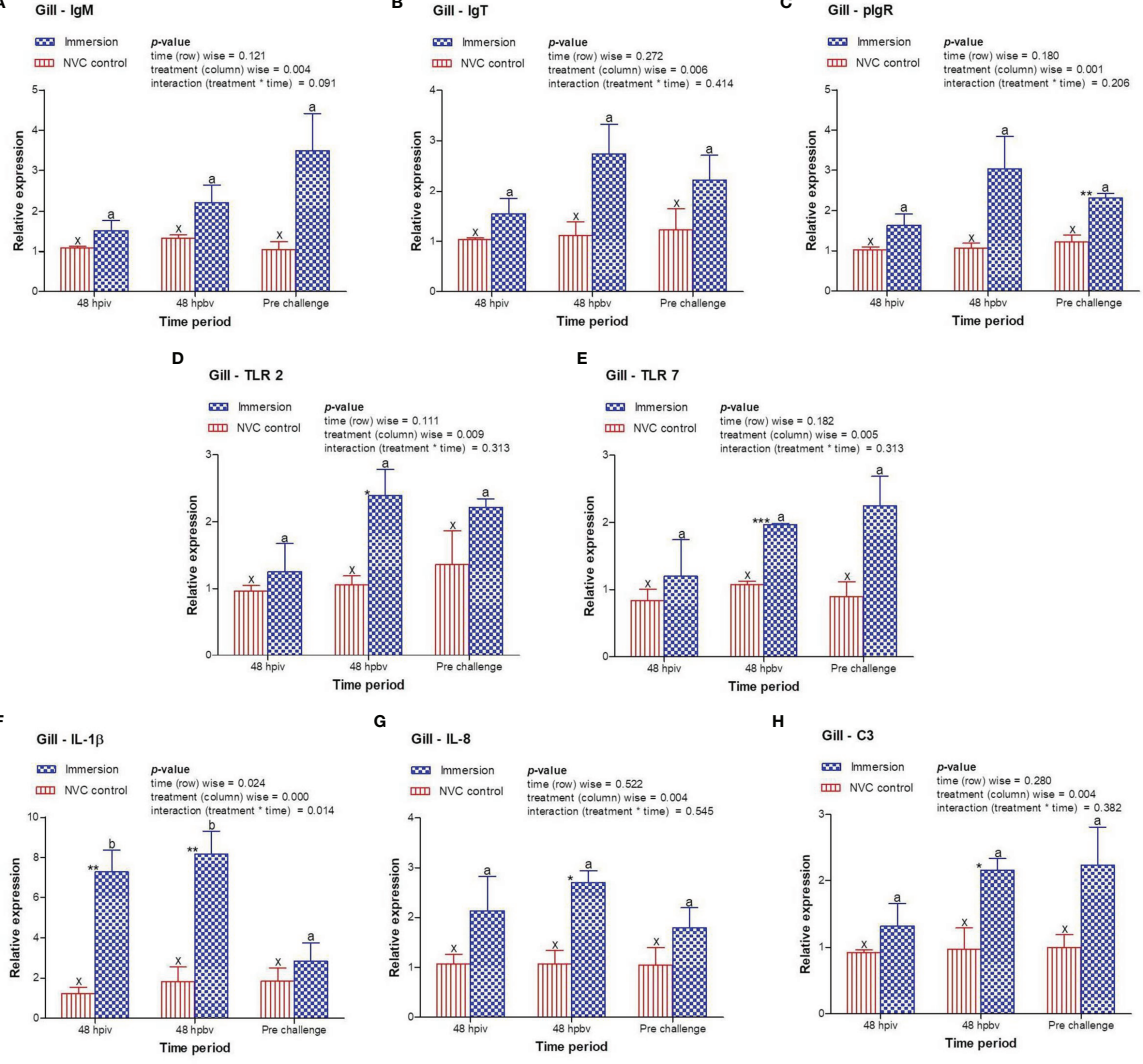
Relative expression of immune genes in gill tissue of olive flounder in the immersion vaccinated and NVC control groups. Expression levels of each gene were compared between the experimental groups relative to the naive control. The mean (*n* = 3) relative expression levels of IgM **(A)**, IgT **(B)**, pIgR **(C)**, TLR 2 **(D)**, TLR 7 **(E)**, IL-1β **(F)**, IL-8 **(G)**, and C3 **(H)** were plotted with standard error at different time intervals post-immunization with the chitosan–PLGA-encapsulated trivalent vaccine. Two-way ANOVA was performed with each dataset. The *p*-values within the group (time-wise), between groups (treatment-wise), and for the interaction effect were evaluated to determine statistical significance (*p* < 0.05). Duncan’s multiple range test [homogenous subsets indicated by lowercase (x-z) for NVC control and (a-c) for Immersion group] within the groups (time-wise) and an unpaired *t*-test (indicated by asterisks * for significance level) between the groups at different time points were also performed to analyze the statistical differences. * denotes significance level of t-test between the 2 groups viz., *, **, *** are for p < 0.05 , 0.01 and 0.001, respectively.

**Figure 5:**
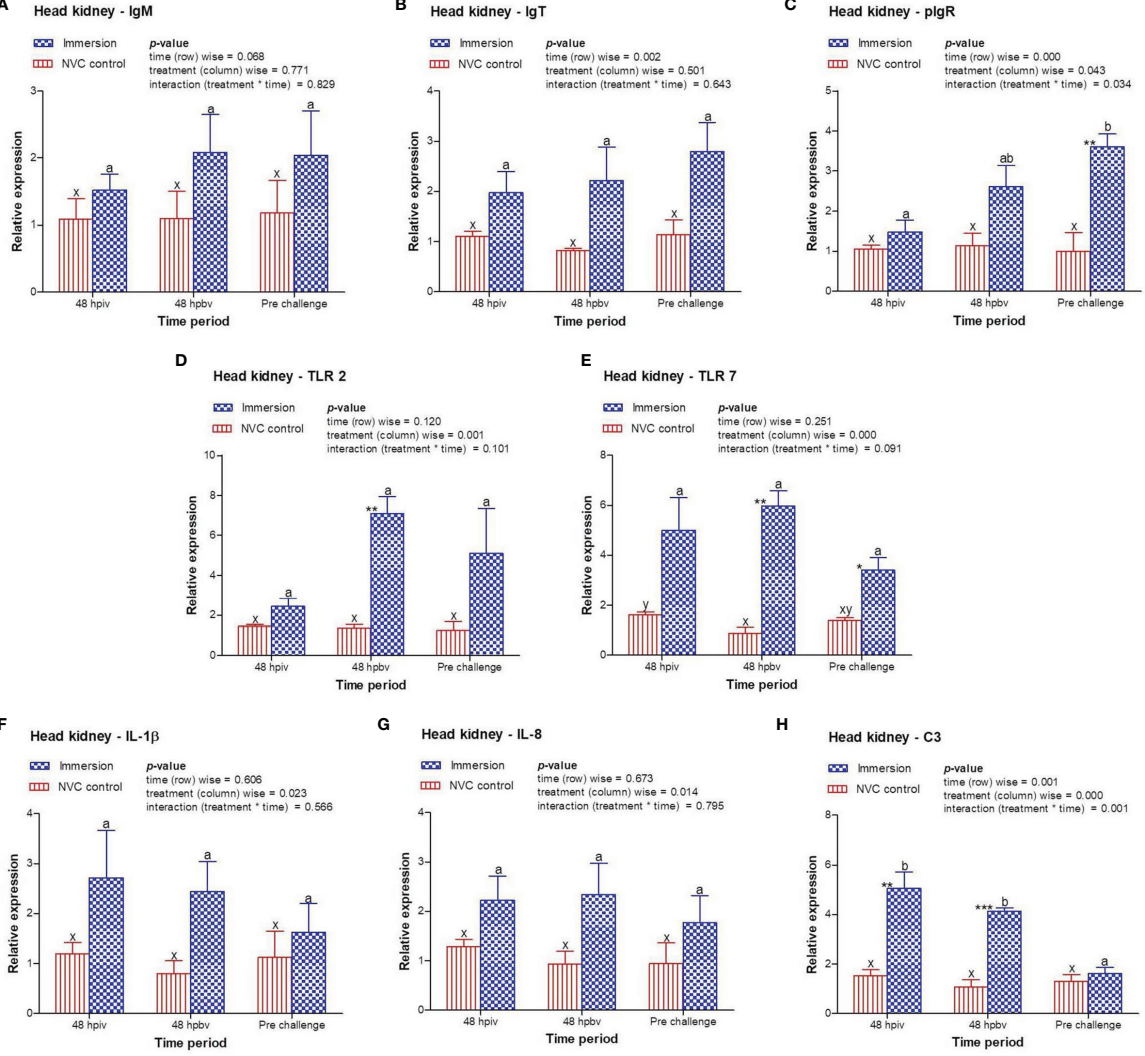
Relative expression of immune genes in anterior kidney tissue of olive flounder in the immersion vaccinated and NVC control groups. Expression levels of each gene were compared between the experimental groups relative to the naive control. The mean (*n* = 3) relative expression levels of IgM **(A)**, IgT **(B)**, pIgR **(C)**, TLR 2 **(D)**, TLR 7 **(E)**, IL-1β **(F)**, IL-8 **(G)**, and C3 **(H)** were plotted with standard error at different time intervals post-immunization with the chitosan–PLGA-encapsulated trivalent vaccine. Two-way ANOVA was performed with each dataset. The *p*-values within the group (time-wise), between groups (treatment-wise), and for the interaction effect were evaluated to determine statistical significance (*p* < 0.05). Duncan’s multiple range test [homogenous subsets indicated by lowercase (x-z) for NVC control and (a-c) for Immersion group] within the groups (time-wise) and an unpaired *t*-test (indicated by asterisks * for significance level) between the groups at different time points were also performed to analyze the statistical differences. * denotes significance level of t-test between the 2 groups viz., *, **, *** are for p < 0.05 , 0.01 and 0.001, respectively.

**Figure 6:**
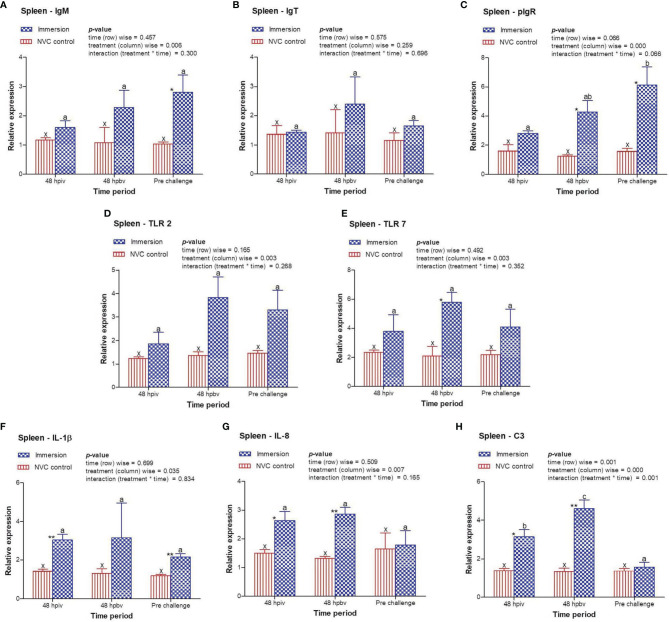
Relative expression of immune genes in spleen tissue of olive flounder in the immersion vaccinated and NVC control groups. Expression levels of each gene were compared between the experimental groups relative to the naive control. The mean (*n* = 3) relative expression levels of IgM **(A)**, IgT **(B)**, pIgR **(C)**, TLR 2 **(D)**, TLR 7 **(E)**, IL-1β **(F)**, IL-8 **(G)**, and C3 **(H)** were plotted with standard error at different time intervals post-immunization with the chitosan–PLGA-encapsulated trivalent vaccine. Two-way ANOVA was performed with each dataset. The *p*-values within the group (time-wise), between groups (treatment-wise), and for the interaction effect were evaluated to determine statistical significance (*p* < 0.05). Duncan’s multiple range test [homogenous subsets indicated by lowercase (x-z) for NVC control and (a-c) for Immersion group] within the groups (time-wise) and an unpaired *t*-test (indicated by asterisks * for significance level) between the groups at different time points were also performed to analyze the statistical differences. ** denotes p < 0.01.

To determine immune induction by the three antigens of the trivalent vaccine, we investigated the expression kinetics of various immune genes both at the skin and gill mucosal surfaces (immersion sites) as well as in the systemic tissues of the head kidney and spleen post-immersion vaccination. The gene expression profiles revealed that in the skin mucosa (**Figure 3**) of the immunized fish, primary immunization (48 hpiv) resulted in a strong and significant (*p* < 0.05) increase in the transcript levels of IgM (2.76-fold), IgT (3.86-fold), pIgR (6.26-fold), TLR 2 (2.25-fold), TLR 7 (1.82-fold), IL-1β (2.65-fold), IL-8 (2.65-fold), and C3 (1.86-fold) when compared to those in NVC control fish; however, at 48 hpbv, there was a decline in the transcript levels of all genes, which further decreased to the control levels at pre-challenge. Conversely, in the gill mucosa (**Figure 4**), except IL-1β (5.86-fold at 48 hpiv), all other analyzed immune genes showed upregulation (~2- to 3-fold) only after booster vaccination, with significant (*p* < 0.05) enhancement in the transcript levels of TLR 2, TLR 7, IL-8, and C3 at 48 hpbv and in pIgR at pre-challenge as compared with the NVC control fish. Likewise, the systemic tissues of the head kidney (**Figure 5**) and spleen (**Figure 6**) in the immunized fish displayed steady upregulation (~2- to 5-fold) in the transcript levels of most analyzed immune genes post-booster vaccination, with the exception of IL-1β and IL-8, which exhibited significant (*p* < 0.05) increases in the gene transcripts post-primary immunization. For C3 gene, transcript levels were significantly upregulated (*p* < 0.05) in the head kidney (3.36- to 3.84-fold) and spleen (2.26- to 3.44-fold) of the immunized fish after both initial and booster vaccination when compared to those of control fish.

### 3.4 Expression Kinetics of Immune-Related Genes Post-Challenge

To elucidate the probable mechanisms behind the observed protective efficacy against individual pathogens in immunized fish, we further analyzed the kinetics of immunoglobulin (Ig) genes and other important immune genes in the anterior kidney and spleen of experimental fish post-challenge with VHSV (**Table 1**), *S. parauberis* type I (**Table 2**), and *M. avidus* (**Table 3**). Expression analysis revealed that all three Ig genes (IgM, IgT, and pIgR) and IL-1β were significantly (*p* < 0.05) upregulated in the vaccinated group in both tissues post-challenge with VHSV and *S. parauberis* type I. However, the differences in induction of the four genes between the immunized and NVC fish groups were much higher in the VHSV-challenged fish than in *S. parauberis* type I-challenged fish. Apart from Ig genes, other innate immune genes, namely, TLR 7, IFN-γ, Mx, and caspase 3 for VHSV and TLR 2, IFN-γ, and caspase 1 for *S. parauberis* type I, also displayed significantly (*p* < 0.05) higher upregulation in the immunized fish than in NVC control fish. In contrast, *M. avidus* challenge resulted in a modest increase in transcripts level of most of the genes, with significant (*p* < 0.05) differences between the immunized and NVC control groups observed only for IgT (at 48–96 hpc) and TNF-α (24 hpc) in the head kidney and for IL-1β (24 hpc), CD-8α (48 hpc), TNF-α (96 hpc), and caspase 3 (24–96 hpc) in the spleen. Another interesting result was the significant increase in the anti-inflammatory IL-10 gene transcripts in both the tissues of NVC control fish post-scuticociliate infection compared to the unaltered transcript level in the immersion group.

**Table 1:** Relative expression of immune genes in anterior kidney and spleen tissues of olive flounder in the immersion vaccinated and NVC control groups at different time intervals post-challenge with virulent VHSV (10^5.8^ TCID_50_ virus/fish).

		Head kidney			Spleen		
		24 hpc	48 hpc	96 hpc	24 hpc	48 hpc	96 hpc
IgM	NVC control	0.92 ± 0.30^x^	1.04 ± 0.09^x^	0.99 ± 0.11^x^	0.86 ± 0.06^x^	0.56 ± 0.10^x^	0.71 ± 0.15^x^
	Immersion	1.72 ± 0.08^a^	*6.65 ± 1.63^b^	5.63 ± 1.70^ab^	2.34 ± 0.67^a^	**2.25 ± 0.29^a^	2.03 ± 0.67^a^
		*p*-value: time—0.054; treatment—0.001; interaction—0.067			*p*-value: time—0.835; treatment—0.001; interaction—0.903		
IgT	NVC control	13.04 ± 3.17^x^	14.58 ± 3.43^x^	8.39 ± 3.02^x^	4.81 ± 1.20^x^	4.84 ± 1.87^x^	3.70 ± 1.31^x^
	Immersion	*63.30 ± 17.46^a^	**103.19 ± 17.56^a^	*90.84 ± 18.87^a^	*13.91 ± 2.83^a^	*16.21 ± 2.31^a^	**11.24 ± 0.96^a^
		*p*-value: time—0.310; treatment—0.000; interaction—0.316			*p*-value: time—0.293; treatment—0.000; interaction—0.601		
pIgR	NVC control	1.83 ± 0.18^x^	12.94 ± 2.87^y^	6.09 ± 1.62^x^	3.68 ± 1.53^x^	6.26 ± 1.78^x^	10.11 ± 3.17^x^
	Immersion	**26.47 ± 4.64^a^	71.34 ± 21.24^a^	*65.49 ± 18.66^a^	*25.23 ± 7.33^a^	**31.18 ± 4.14^a^	55.84 ± 19.19^a^
		*p*-value: time—0.082; treatment—0.000; interaction—0.281			*p*-value: time—0.125; treatment—0.001; interaction—0.355		
IL-1β	NVC control	0.46 ± 0.23^x^	20.04 ± 3.32^y^	5.75 ± 1.40^x^	7.71 ± 1.06^x^	111.20 ± 42.64^y^	6.69 ± 2.17^x^
	Immersion	**7.99 ± 1.24^a^	**68.74 ± 9.79^b^	*12.24 ± 1.46^a^	29.51 ± 9.98^a^	*251.30 ± 12.02^b^	42.34 ± 14.09^a^
		*p*-value: time—0.000; treatment—0.000; interaction—0.000			*p*-value: time—0.005; treatment—0.001; interaction—0.000		
IFN-γ	NVC control	7.16 ± 2.91^x^	194.43 ± 17.89^y^	26.55 ± 6.72^x^	18.57 ± 5.14^x^	130.69 ± 12.17^y^	14.29 ± 2.20^x^
	Immersion	24.24 ± 7.77^a^	*610.32 ± 130.07^b^	43.71 ± 6.85^a^	23.49 ± 2.02^a^	*247.58 ± 22.35^b^	*36.08 ± 6.05^a^
		*p*-value: time—0.000; treatment—0.005; interaction—0.004			*p*-value: time—0.000; treatment—0.000; interaction—0.001		
Mx	NVC control	1.06 ± 0.33^x^	94.05 ± 17.99^y^	68.53 ± 17.79^y^	13.63 ± 5.26^x^	159.56 ± 9.12^y^	201.92 ± 51.28^y^
	Immersion	*5.16 ± 0.96^a^	341.53 ± 92.52^b^	*261.19 ± 41.77^b^	35.60 ± 8.65^a^	***574.40 ± 36.72^c^	317.16 ± 41.91^b^
		*p*-value: time—0.001; treatment—0.001; interaction—0.035			*p*-value: time—0.000; treatment—0.000; interaction—0.000		
TLR 7	NVC control	0.74 ± 0.20^x^	2.07 ± 0.45^xy^	2.56 ± 0.67^y^	3.12 ± 1.30^x^	3.72 ± 0.68^x^	3.56 ± 1.60^x^
	Immersion	2.21 ± 0.62^a^	5.61 ± 1.37^a^	5.15 ± 1.57^a^	6.90 ± 2.35^a^	10.36 ± 2.92^a^	8.46 ± 2.78^a^
		*p*-value: time—0.042; treatment—0.007; interaction—0.569			*p*-value: time—0.637; treatment—0.011; interaction—0.793		
Caspase 3	NVC control	1.41 ± 0.21^x^	1.22 ± 0.22^x^	3.16 ± 0.16^y^	0.86 ± 0.28^x^	1.03 ± 0.33^x^	1.86 ± 0.39^x^
	Immersion	*4.14 ± 0.64^a^	**16.25 ± 2.63^b^	**12.58 ± 1.23^b^	*3.05 ± 0.64^a^	*4.38 ± 0.87^a^	6.23 ± 1.83^a^
		*p*-value: time—0.001; treatment—0.000; interaction—0.001			*p*-value: time—0.102; treatment—0.001; interaction—0.504		

Mean (n = 3) expression levels of each gene relative to the naive control are tabulated with standard error at different time intervals. Two-way ANOVA was performed with each dataset, and the p-values within the group (time-wise), between groups (treatment-wise), and for the interaction effect were evaluated to determine statistical significance (p < 0.05). Duncan’s multiple range test (homogenous subsets indicated by lowercase [x-z] for NVC control and [a-c] for Immersion group) within the groups (time-wise) and an unpaired t-test (indicated by asterisks * for significance level) between the groups at different time points were also performed to analyze the statistical differences. * denotes significance level of t-test between the 2 groups viz., *, **, *** are for p < 0.05 , 0.01 and 0.001, respectively.

**Table 2:** Relative expression of immune genes in anterior kidney and spleen tissues of olive flounder in the immersion vaccinated and NVC control groups at different time intervals post-challenge with virulent *S. parauberis* type I (1.6 × 10^9^ CFU/fish).

		Head kidney			Spleen		
		24 hpc	48 hpc	96 hpc	24 hpc	48 hpc	96 hpc
IgM	NVC control	0.95 ± 0.35^x^	0.52 ± 0.11^x^	0.45 ± 0.06^x^	0.89 ± 0.31^x^	1.38 ± 0.50^x^	1.25 ± 0.34^x^
	Immersion	1.37 ± 0.14^a^	1.19 ± 0.38^a^	**1.86 ± 0.26^a^	3.68 ± 1.12^a^	2.65 ± 1.03^a^	2.98 ± 1.06^a^
		*p*-value: time—0.395; treatment—0.001; interaction—0.158			*p*-value: time—0.947; treatment—0.013; interaction—0.638		
IgT	NVC control	1.66 ± 0.43^x^	1.50 ± 0.63^x^	1.68 ± 0.73^x^	2.07 ± 1.11^x^	2.31 ± 1.51^x^	3.28 ± 1.37^x^
	Immersion	2.29 ± 0.39^a^	*4.25 ± 0.07^ab^	*8.90 ± 2.47^b^	3.48 ± 1.17^a^	6.71 ± 1.35^a^	6.70 ± 2.71^a^
		*p*-value: time—0.030; treatment—0.002; interaction—0.033			*p*-value: time—0.389; treatment—0.039; interaction—0.654		
pIgR	NVC control	1.29 ± 0.22^x^	1.12 ± 0.18^x^	1.20 ± 0.28^x^	4.97 ± 0.44^y^	5.22 ± 0.72^y^	2.60 ± 0.27^x^
	Immersion	*2.95 ± 0.41^ab^	2.58 ± 0.69^a^	**4.52 ± 0.42^b^	**10.97 ± 1.08^a^	**10.20 ± 0.51^a^	**13.59 ± 2.07^a^
		*p*-value: time—0.070; treatment—0.000; interaction—0.077			*p*-value: time—0.931; treatment—0.000; interaction—0.029		
IL-1β	NVC control	19.95 ± 1.58^y^	17.22 ± 4.66^y^	3.70 ± 0.89^x^	828.24 ± 57.18^y^	105.87 ± 10.25^x^	11.85 ± 1.29^x^
	Immersion	**80.59 ± 9.59^b^	26.43 ± 3.45^b^	*10.36 ± 2.19^a^	**2874.30 ± 9.98^b^	**358.58 ± 40.80^a^	*39.25 ± 9.13^a^
		*p*-value: time—0.000; treatment—0.000; interaction—0.000			*p*-value: time—0.000; treatment—0.000; interaction—0.000		
TLR 2	NVC control	1.21 ± 0.29^x^	1.08 ± 0.33^x^	1.23 ± 0.52^x^	1.26 ± 0.17^x^	1.01 ± 0.07^x^	0.99 ± 0.14^x^
	Immersion	1.97 ± 0.16^a^	2.02 ± 0.26^a^	2.42 ± 0.43^a^	**3.40 ± 0.20^b^	*2.29 ± 0.30^a^	**2.33 ± 0.18^a^
		*p*-value: time—0.705; treatment—0.006; interaction—0.839			*p*-value: time—0.005; treatment—0.000; interaction—0.074		
IFN-γ	NVC control	3.27 ± 1.17^x^	2.37 ± 0.63^x^	1.71 ± 0.56^x^	4.39 ± 0.67^x^	3.49 ± 1.10^x^	1.92 ± 0.28^x^
	Immersion	**13.58 ± 1.76^b^	*8.37 ± 1.47^a^	*6.49 ± 0.96^a^	***17.36 ± 1.21^c^	5.61 ± 0.13^b^	2.81 ± 0.17^a^
		*p*-value: time—0.009; treatment—0.000; interaction—0.085			*p*-value: time—0.000; treatment—0.000; interaction—0.000		
Caspase 1	NVC control	1.49 ± 0.27^y^	1.05 ± 0.21^xy^	0.70 ± 0.08^x^	25.78 ± 1.90^y^	23.98 ± 5.48^y^	9.41 ± 4.09^x^
	Immersion	2.39 ± 0.42^a^	4.30 ± 1.29^ab^	**6.32 ± 0.88^b^	*33.03 ± 1.37^a^	46.44 ± 10.79^a^	*47.06 ± 7.39^a^
		*p*-value: time—0.107; treatment—0.000; interaction—0.015			*p*-value: time—0.493; treatment—0.001; interaction—0.082		

Mean (n = 3) expression levels of each gene relative to the naive control are tabulated with standard error at different time intervals. Two-way ANOVA was performed with each dataset, and the p-values within the group (time-wise), between groups (treatment-wise), and for the interaction effect were evaluated to determine statistical significance (p < 0.05). Duncan’s multiple range test (homogenous subsets indicated by lowercase [x-z] for NVC control and [a-c] for Immersion group) within the groups (time-wise) and an unpaired t-test (indicated by asterisks * for significance level) between the groups at different time points were also performed to analyze the statistical differences. * denotes significance level of t-test between the 2 groups viz., *, **, *** are for p < 0.05 , 0.01 and 0.001, respectively.

**Table 3:** Relative expression of immune genes in anterior kidney and spleen tissues of olive flounder in the immersion vaccinated and NVC control groups at different time intervals post-challenge with virulent *M. avidus* (5 × 10^4^ cells/fish).

		Head kidney			Spleen		
		24 hpc	48 hpc	96 hpc	24 hpc	48 hpc	96 hpc
IgM	NVC control	1.87 ± 0.22^x^	1.53 ± 0.46^x^	1.44 ± 0.60^x^	1.02 ± 0.28^x^	1.01 ± 0.26^x^	0.90 ± 0.27^x^
	Immersion	2.56 ± 1.18^a^	2.05 ± 0.66^a^	2.16 ± 0.81^a^	1.24 ± 0.45^a^	2.64 ± 0.67^a^	1.80 ± 0.65^a^
		*p*-value: time—0.798; treatment—0.292; interaction—0.988			*p*-value: time—0.341; treatment—0.033; interaction—0.345		
IgT	NVC control	0.28 ± 0.12^x^	0.42 ± 0.26^x^	2.20 ± 0.90^x^	3.20 ± 1.54^x^	2.73 ± 1.17^x^	2.29 ± 0.76^x^
	Immersion	1.87 ± 1.02^a^	*23.71 ± 8.30^b^	*20.05 ± 5.47^ab^	3.97 ± 1.19^a^	4.66 ± 1.88^a^	4.96 ± 1.70^a^
		*p*-value: time—0.036; treatment—0.001; interaction—0.053			*p*-value: time—0.997; treatment—0.149; interaction—0.801		
pIgR	NVC control	2.84 ± 0.93^x^	3.80 ± 1.02^x^	2.87 ± 0.59^x^	1.57 ± 0.17^x^	2.31 ± 0.74^x^	2.64 ± 0.70^x^
	Immersion	5.30 ± 1.57^a^	5.19 ± 0.84^a^	4.26 ± 1.92^a^	2.98 ± 0.71^a^	4.50 ± 1.42^a^	5.47 ± 1.34^a^
		*p*-value: time—0.755; treatment—0.108; interaction—0.882			*p*-value: time—0.207; treatment—0.017; interaction—0.762		
IL-1β	NVC control	2.29 ± 0.11^x^	2.38 ± 0.29^x^	2.45 ± 0.02^x^	0.87 ± 0.26^x^	1.30 ± 0.28^x^	1.14 ± 0.35^x^
	Immersion	3.42 ± 0.67^a^	*4.18 ± 0.32^a^	3.54 ± 0.82^a^	*2.20 ± 0.40^a^	2.37 ± 0.48^a^	2.70 ± 0.63^a^
		*p*-value: time—0.659; treatment—0.004; interaction—0.702			*p*-value: time—0.633; treatment—0.002; interaction—0.845		
IL-10	NVC control	2.76 ± 1.30^x^	3.43 ± 1.53^x^	3.40 ± 0.89^x^	1.62 ± 0.40^x^	3.18 ± 0.52^xy^	3.96 ± 0.89^y^
	Immersion	1.55 ± 0.65^a^	1.73 ± 0.27^a^	1.58 ± 0.31^a^	1.30 ± 0.10^b^	**0.73 ± 0.10^a^	*1.24 ± 0.13^b^
		*p*-value: time—0.895; treatment—0.065; interaction—0.945			*p*-value: time—0.081; treatment—0.000; interaction—0.043		
TNF-α	NVC control	2.01 ± 0.15^x^	2.24 ± 0.57^x^	1.77 ± 0.52^x^	1.29 ± 0.14^x^	1.09 ± 0.14^x^	1.12 ± 0.10^x^
	Immersion	*3.39 ± 0.32^b^	2.10 ± 0.08^a^	2.81 ± 0.45^ab^	1.73 ± 0.22^a^	2.24 ± 0.47^a^	**1.80 ± 0.08^a^
		*p*-value: *time—*0.394; treatment—0.036; interaction—0.172			*p*-value: time—0.656; treatment—0.002; interaction—0.328		
IL-8	NVC control	2.28 ± 0.27^x^	3.45 ± 0.40^x^	4.03 ± 1.14^x^	2.60 ± 0.39^x^	3.78 ± 0.19^x^	3.32 ± 0.97^x^
	Immersion	3.84 ± 0.55^a^	4.95 ± 1.38^a^	5.16 ± 1.64^a^	3.17 ± 0.63^a^	7.95 ± 2.34^a^	6.59 ± 1.70^a^
		*p*-value: *time—*0.338; treatment—0.124; interaction—0.975			*p*-value: time—0.098; treatment—0.026; interaction—0.377		
CD-8α	NVC control	1.87 ± 0.49^x^	1.70 ± 0.36^x^	1.75 ± 0.14^x^	0.64 ± 0.18^x^	1.38 ± 0.24^xy^	1.55 ± 0.24^y^
	Immersion	2.33 ± 0.68^a^	2.39 ± 0.65^a^	2.60 ± 0.52^a^	1.09 ± 0.65^a^	**2.98 ± 0.24^b^	2.48 ± 0.49^ab^
		*p*-value: time—0.968; treatment—0.133; interaction—0.929			*p*-value: time—0.009; treatment—0.008; interaction—0.349		
Caspase 3	NVC control	1.39 ± 0.25^x^	1.47 ± 0.55^x^	1.06 ± 0.25^x^	0.87 ± 0.08^x^	1.70 ± 0.30^y^	2.81 ± 0.18^z^
	Immersion	1.38 ± 0.26^a^	2.10 ± 0.37^a^	1.90 ± 0.46^a^	**1.35 ± 0.03^a^	3.63 ± 1.01^b^	*4.05 ± 0.39^b^
		*p*-value: time—0.553; treatment—0.139; interaction—0.514			*p*-value: time—0.001; treatment—0.008; interaction—0.325		

Mean (n = 3) expression levels of each gene relative to the naive control are tabulated with standard error at different time intervals. Two-way ANOVA was performed with each dataset, and the p-values within the group (time-wise), between groups (treatment-wise), and for the interaction effect were evaluated to determine statistical significance (p < 0.05). Duncan’s multiple range test (homogenous subsets indicated by lowercase [x-z] for NVC control and [a-c] for Immersion group) within the groups (time-wise) and an unpaired t-test (indicated by asterisks * for significance level) between the groups at different time points were also performed to analyze the statistical differences. * denotes significance level of t-test between the 2 groups viz., * and ** are for p < 0.05 and 0.01 respectively.

## 4 Discussion

Among the 28 commercialized vaccines approved by the Korean government for olive flounder against various diseases to date, most are injection-based, and only two are delivered *via* immersion (26). However, the current disease status of olive flounders indicates that young small fingerlings, for which injection vaccines are highly stressful and non-feasible, are much more susceptible throughout their culture cycle to different diseases, such as VHSV, scuticociliatosis, and streptococcosis, resulting in high losses to the flounder industry. Thus, we developed a trivalent formalin-killed vaccine containing VHSV, *S. parauberis* type I, and *M. avidus* antigens and encapsulated it in chitosan–PLGA nanoparticles. After development, the vaccine was administered to olive flounder fingerlings *via* immersion involving a prime-boost immunization strategy, which were subsequently challenged with the three targeted pathogens to evaluate the efficacy and potency of the vaccine in stimulating protective immune responses in the host.

RPS analysis revealed that the immunized fish were moderately immune to all three pathogens, with 66.63%, 53.3%, and 66.75% RPS against VHSV, *S. parauberis* type I, and *M. avidus* challenges, respectively. The vaccine efficacy of 66.63% against VHSV is in line with our previous studies (16, 17), where we observed 60%–73% RPS in olive flounder immunized with a chitosan/PLGA-encapsulated monovalent VHSV vaccine; however, it is comparatively lower than the recorded protective efficacy (72%–89% RPS) of the immersion vaccine developed by Hwang et al. (27), who used Montanide IMS 1312 VG adjuvant with heat-inactivated VHSV. However, given the trivalent nature of our vaccine, the result is quite promising for countering VHSV infection in olive flounder farming. Regarding protective efficacy against *S. parauberis* type I, although the observed RPS of 53.3% is much lower than the results of previous studies at 75% RPS (4) and 100% RPS (28), it is important to consider here that both the studies involved administration of an inactivated vaccine *via* highly stressful intraperitoneal injection route as compared to the present non-stressful immersion route. To the best of our knowledge, as there are no reported studies regarding a mucosal vaccine against *S. parauberis*, the current immersion vaccination strategy yielding moderate efficacy is greatly helpful for future development of more efficient mucosal vaccines against *S. parauberis* type I for olive flounder. Considering the vaccine efficacy against *M. avidus*, we previously investigated an injected naked formalin-killed vaccine olive flounder immunization, which failed to exhibit any protection against the parasite (29). However, in the present study, as we encapsulated the FKC ciliate antigen with chitosan–PLGA particles and immunized using the immersion route, good efficacy (66.75% RPS) was obtained. Thus, it can be said that the encapsulation of the scuticociliate antigen with chitosan–PLGA particles positively influenced the targeted delivery of the vaccine *via* skin and gill surfaces (natural infection route for parasites), in turn triggering protective immune responses against the parasite. Similar observations were also reported in a study involving a microsphere-based scutica vaccine in turbot, where a chitosan-based microsphere for scuticociliate antigen elicited moderate (58%–60% RPS) protection (30), which supports our vaccination strategy against scuticociliates.

In addition to vaccine efficacy analysis, the current study quantified the specific antibody titers against VHSV, *S. parauberis* type I, and *M. avidus* post-vaccination and at 48 hpc. The anti-VHSV antibody titer in serum demonstrated an increasing trend from 48 hpiv in the immersion group, which remained significantly (*p* < 0.05) higher than that of the NVC control group throughout the immunization period, with further enhancement of the titer at 48 hpc with VHSV. These antibody titers can be correlated with the protection against VHSV challenge (RPS %) in the vaccinated fish when compared with the NVC control group, indicating that our vaccine was effective in stimulating the humoral immune response as well as production of anti-VHSV Ig. Moreover, the relatively low titers in the vaccinated fish obtained at various time points compared to those in our previous studies involving a monovalent VHSV vaccine (16, 17) might be due to antigen competition in stimulating specific antibodies, resulting in a reduction in the maximal antibody responses against individual pathogens in the immunized fish. Similarly, the anti-*S. parauberis* type I antibody titers in the immersion group also showed incremental increases post-immunization and were significantly (*p* < 0.05) higher than those of the NVC control group. The observed antibody titers in immunized fish serum were relatively lower than those previously reported for *S. parauberis* vaccination in olive flounder (4, 28), where the injection route was employed. Furthermore, the result regarding quantification of anti-*M. avidus* antibody revealed a steady and significant (*p* < 0.05) increase in titers in the vaccinated group compared to the NVC control group post-immunization. The increase in anti-*M. avidus* antibody titers obtained after immersion vaccination is quite encouraging, as similar results have been observed in studies of injection-based scuticociliate vaccines in flounder (29, 31) and turbot (30, 32–35), in which whole (formalin-killed), lysed, or other ciliate components (cilia or membrane) were used as the vaccine antigen. It is pertinent to mention here that the sharp rise in antibody titer in the NVC control group after *M. avidus* challenge, which narrowed the difference in titers between the two experimental groups to an insignificant (*p* > 0.05) level, might be the reason behind the relatively lower mortality in NVC control fish compared to that in immunized fish. This is in accordance with the parasite-induced enhancement of antibody titer in *Ichthyophthirius multifiliis* (Ich) parasite-infected rainbow trout (36). Moreover, the high antibody responses at 48 hpc against all the three pathogens can be explained by the fact that we employed c-ELISA to quantify the anti-pathogen humoral responses present in the sera, which were not only limited to the IgM responses but the sum total of anti-pathogen Ig responses (IgM/IgT) as well as other humoral factors; as IgT responses are much quicker unlike IgM (as observed from the strong induction of IgT gene expression post challenge in the systemic tissues), the high PI values can be correlated with it. Similar observations of IgT antibody response alongside IgM response in the serum of immunized Gilthead sea bream were observed post challenge with the parasite *Enteromyxum leei*, *Photobacterium damselae* subsp. *Piscicida*, and nodavirus (37). Therefore, considering the antibody titers and RPS, it can be inferred that our vaccine had a positive effect in stimulating humoral immunity of the vaccinated fish, thus providing protection against each of the three virulent pathogens.

In absence of standardized fish vaccine correlates, the expression kinetics of immune genes are generally employed for determining correlations in fish vaccine potency (38). In view of this, we included expression profiling of different classes of immune-related genes in the mucosal and systemic tissues of the experimental fish post-immersion immunization with the trivalent vaccine. Exposure to vaccine antigen leads to creation of the immunological memory, which elicits an enhanced response on subsequent encounters with the same antigen, resulting in adaptive immunity. The humoral components of the teleost adaptive immunity consist of Igs molecules, secreted from the plasma cells into blood and mucus, and are important parameters for evaluating vaccine potency (21, 39, 40). The present results showed a strong and significant (*p* < 0.05) increase in the transcript levels of IgM, IgT, and their receptor pIgR in the skin mucosa of immunized fish post-primary vaccination, after which the expression levels declined, whereas in gill mucosa and in the systemic tissues of the head kidney and spleen, all three genes steadily increased starting at 48 hpiv with significant (*p* < 0.05) upregulation recorded at 48 hpbv to the pre-challenge period compared to the control fish. Although this result does not give exact details regarding the simultaneous release of the three antigens and their individual contributions to Ig stimulation, the overall expression patterns can be correlated with the potency of our trivalent vaccine in inducing production of Ig-secreting cells in both the mucosal and systemic immune compartment of the immunized fish and facilitating antibody-dependent humoral immunity.

In addition, the current study investigated the expression profile of genes involved in toll-like receptor (TLR) pathways, such as TLR 2 and TLR 7 for *S. parauberis* type I and VHSV antigens, respectively. Similar to Ig expression, both TLR 2 and TLR 7 genes were significantly (*p* < 0.05) upregulated in the skin tissue post-initial vaccination, whereas in the gills, head kidney, and spleen, significant (*p* < 0.05) upregulation of the gene transcripts were observed post-booster dose with respect to those of the NVC control group. These increases in TLR 2 and TLR 7 gene transcript levels in the mucosal and systemic tissues of the immunized fish confirm the utility of nanoencapsulation for targeted delivery of the antigens to receptor cells, which may facilitate subsequent recognition and binding to induce effective innate immune responses against bacterial and viral antigens. Furthermore, we evaluated the expression of the pro-inflammatory cytokine IL-1β and CXC chemokine IL-8. Unlike Igs and TLRs, both IL-1β and IL-8 genes showed significant (*p* < 0.05) upregulation in transcript levels post-primary immunization in both the mucosal and systemic tissues of immunized fish compared with those in the control, indicating the induction of inflammatory response post-immunization, which helps in initiating other innate immune pathways (27, 41, 42). In addition, we examined the expression kinetics of complement factor C3, as the complement system plays an integral part in host defense irrespective of pathogen type (43). A significant (*p* < 0.05) increase in the C3 transcript level was observed in both the mucosal and systemic tissues of immunized fish post-primary and post-booster vaccination. This increase in the C3 transcript level post-vaccination indicates that the vaccine antigens (one, two, or all three) initiated the complement pathway, thus triggering the innate immunity in the host, which in turn may help in establishing immunological memory against different antigens to counter subsequent infection with the respective pathogens.

To determine correlations between the vaccine potency and the counteractive immune mechanisms against the three pathogens, we further examined the kinetics of important immune genes related to each pathogen in addition to adaptive immunity-related genes in the head kidney and spleen tissues of experimental fish post-intraperitoneal injection challenge with VHSV, *S. parauberis* type I, and *M. avidus*.

From the expression kinetics of immune genes analyzed post-VHSV challenge, it was observed that IgM gene transcripts were upregulated in the vaccinated group at all time points in both head kidney and spleen tissues, with significant (*p* < 0.05) differences observed at 48 hpc compared to the NVC control group, which showed no changes in transcript level. Unlike IgM, the IgT and pIgR gene transcripts were enhanced in both immunized and NVC control groups post-challenge, but the increase in vaccinated fish was much greater than that in NVC control fish. The Ig expression post-VHSV challenge is consistent with the findings of our previous studies with monovalent vaccines (16, 17), substantiating the definite role of the nanoencapsulated vaccine in channeling the adaptive humoral response against the virus. Notably, although the expression pattern for IgT in the head kidney coincides with our previous findings, the wide differences in the fold change was due to variation in the lower basal expression of IgT (Ct value ~35 compared to 37 previously) in the naive control fish. High and significant (*p* < 0.05) upregulation of IL-1β was also observed at early stages of infection in both tissues in the Immersion group compared to those in NVC control fish, suggesting a proactive inflammatory response in the immunized fish for combating the virus. Moreover, the study revealed a two- to threefold higher increase in the gene transcript level of TLR 7 [of pattern recognition receptor (PRR)-mediated innate response] in the vaccinated fish than in the NVC control fish in both the kidney and spleen, indicating improved recognition of virus-derived ssRNA antigens and subsequent induction of different downstream antiviral pathways (44, 45). Other than PRR-mediated response for determining the antiviral potency of viral vaccines in fish, analysis of the transcript changes in interferon-gamma (IFN-γ) and Mx genes are important correlates, as both these genes in their own way can help in restricting the overall proliferation of the VHSV infection; IFN-γ by triggering Th1 responses to induce cell-mediated cytotoxicity, and Mx by suppressing viral replication in infected cells (46–48). Thus, we analyzed the expression of IFN-γ and Mx genes. Both the vaccinated and NVC control fish (in the head kidney and spleen tissues) showed significant (*p* < 0.05) upregulation in IFN-γ and Mx transcript levels post-VHSV challenge, with the peak observed at 48 hpc, but the recorded transcript levels of both genes were approximately three to four times higher in the Immersion group than in the NVC control group, indicating a much stronger and transient T-cell-mediated cytotoxicity in the immunized fish, which ultimately resulted in improved survival. In addition, the study demonstrated significantly (*p* < 0.05) high induction of caspase 3, the final mediator of cell death (49–51), in the vaccinated fish at all the time points post-challenge compared to that of NVC control fish, reflecting a rapid apoptotic reaction in the vaccinated host, which may be due to T-cell-mediated or through perforin/granzyme pathway or by the virus itself, but in any case, the timely apoptotic reaction indicated by strong caspase 3 gene expression has helped in limiting viral proliferation, leading to improved protection against VHSV.

Our results of immune gene expression post-challenge with *S. parauberis* type I exhibited an increasing expression pattern for IgM, IgT, and pIgR genes at 24–96 hpc in the vaccinated group only, whereas in the NVC control group, the transcript levels remained in a subdued and static condition at all time points. These significant (*p* < 0.05) differences in Ig genes between the immunized and NVC control groups post-bacterial challenge can be correlated with their survival percentages, thus suggesting a key role of the vaccine-induced humoral immune responses in protecting the immunized fish against the bacteria. Apart from Igs, other innate immune genes, such as IL-1β, TLR 2, IFN-γ, and caspase 1, were also significantly (*p* < 0.05) upregulated in the immunized fish compared to those in NVC control fish. This finding further confirmed that our vaccine is effective in orchestrating an anti-bacterial defense mechanism in the immunized host, thus reducing the bacterial load and preventing severe pathogenesis. Moreover, the spleen tissue displayed an overall higher induction of the gene transcripts than the head kidney exhibited at all time points post-challenge with *S. parauberis* type I, indicating that the spleen is an important defense organ for fighting bacteria; however, the exact cause needs further investigation. Nevertheless, in the absence of relevant studies regarding gene expression post-vaccination against *S. parauberis* type I, our results provide critical baseline data for future works, substantiating the importance of our study.

Following *M. avidus* challenge, overall moderate expression levels of most immune genes were observed in both experimental fish groups. The specific antibodies generating Ig’s gene, i.e., IgM, IgT, and pIgR, showed higher transcript levels in the immunized fish than in the NVC control; however, the fold-change differences were not significant (*p* > 0.05), barring the distinctive upregulation of IgT (*p* < 0.05) noticed in the head kidney at 48–96 hpc. In addition, the gene transcript levels of the complement system (innate humoral response), including complement factors D and 3, C1q, and C-type lectin, exhibited similar increases in both experimental groups with insignificant fold-change differences between them (data not shown). Although these results can be attributed to improved protection in the immunized fish against scuticociliate challenge, they failed to provide conclusive evidence of the efficacy of our vaccine in triggering the humoral (innate or adaptive) immune responses against the parasite in the host. Moreover, the non-significant difference in the transcript levels between the immunized and control fish post-challenge can be explained by previous studies involving natural infection of ciliate in turbot, which reported that non-sensitized naive fish after countering the ciliates were able to neutralize them and release proteolytic protease, which in turn helps in generating specific antibodies (32) and complement components (52). Likewise, it was observed that the transcript levels of pro-inflammatory cytokine and chemokine genes (IL-1β, TNF-α, and IL-8) were higher in the vaccinated fish than in the NVC controls at all time points post-challenge, but distinct and significant (*p* < 0.05) fold-change differences were only noticed in the head kidney for TNF-α at 24 hpc and IL-1β at 48 hpc. Similar results were observed by several previous authors, who reported very weak or absent inflammatory reactions in fish following scuticociliate infection (53–55). Moreover, the significant increase in gene transcripts of the anti-inflammatory cytokine IL-10 in both tissues of NVC control fish compared with unchanged transcript levels in the immunized fish can be correlated with the immune evasion property of scuticociliates in bypassing the host inflammatory response (56, 57), which might have led to increased pathogenicity and mortality in the NVC control group. Furthermore, our results also revealed significantly (*p* < 0.05) higher upregulation of CD-8α and caspase 3 genes in the spleen of immunized fish compared to those in NVC controls, suggesting a probable T-cell-dependent apoptotic reaction in the parasite-infected cells leading to a reduction in parasite load. However, the exact mechanism responsible for improved survival in vaccinated fish as well as overall lower mortality in both experimental groups post-scuticociliate challenge requires detailed investigation in the future.

In conclusion, our study revealed that immersion delivery of a chitosan–PLGA-encapsulated trivalent vaccine is an effective immunization strategy for protection against three important diseases in olive flounder. From the immunization study, it was evident that the use of chitosan–PLGA for nanoencapsulation of the three antigens facilitated non-stressful administration to the targeted immune organs, which in turn helped in orchestrating pathogen-specific immune responses against VHSV, *S. parauberis* type I, and *M. avidus*. Our study is also unique and of significance because it provides critical insights and baseline information about the immune response post-immersion vaccination against *S. parauberis* type I and *M. avidus*. Thus, it can be inferred that the formulated trivalent mucosal vaccine can protect olive flounder throughout its culture cycle, and with further refinement, the vaccination strategy can become an important prophylactic measure in the flounder industry against VHS, streptococcosis, and scuticociliatosis diseases.

## Data Availability Statement

The raw data supporting the conclusions of this article will be made available by the authors, without undue reservation.

## Ethics Statement

The present study was conducted in accordance with the recommendations by the Institutional Animal Care and Use Committee (IACUC) of Chonnam National University (CNUIACUC-YS-2018-3), which is in regular compliance with the animal protection, welfare, and research, under Article 13 of the Animal Protection Act and Article 7 of the Enforcement Degree of the Act, Republic of Korea.

## Author Contributions

SK designed the study, performed experiments, analyzed data, and wrote the original manuscript. S-MS performed experiments. SAD and H-JJ analyzed data and prepared the manuscript. S-JJ conceived ideas, designed the study, oversaw research, and reviewed and edited the manuscript. All authors contributed to the article and approved the submitted version.

## Funding

This research was supported by the Fish Vaccine Research Center funded by the Ministry of Oceans and Fisheries, Republic of Korea.

## Conflict of Interest

The authors declare that the research was conducted in the absence of any commercial or financial relationships that could be construed as a potential conflict of interest.

## Publisher’s Note

All claims expressed in this article are solely those of the authors and do not necessarily represent those of their affiliated organizations, or those of the publisher, the editors and the reviewers. Any product that may be evaluated in this article, or claim that may be made by its manufacturer, is not guaranteed or endorsed by the publisher.
