# Supplementary material for: Potential Efficacy of Chitosan-Poly (Lactide-Co-Glycolide)-Encapsulated Trivalent Immersion Vaccine in Olive Flounder (Paralichthys olivaceus) Against Viral Hemorrhagic Septicemia Virus, Streptococcus parauberis Serotype I, and Miamiensis avidus (Scuticociliate)
Source: Front Immunol. 2021 Dec 2;12:761130. doi: 10.3389/fimmu.2021.761130 (PMC8677005; doi:10.3389/fimmu.2021.761130)
Supplement: Supplementary file 1 [file DataSheet_1.docx]

Supplementary Material

# Competitive ELISA for quantification of specific antibody against VHSV

Specific antibody (anti-VHSV Ig) quantification in the fish sera of the experimental fish groups was done by competitive enzyme-linked immunosorbent assays (c-ELISA) according to the protocol of Kole et al. (16) with necessary modifications. Briefly, 100 µL of VHSV (10^8^ TCID_50_/mL) diluted in coating buffer (carbonate–bicarbonate buffer, pH 9.6) was coated in triplicate in 96-well ELISA plates (Nunc, Denmark) and incubated at 4 °C overnight. The plate was washed with wash buffer (PBS-T, 0.05% tween20 in PBS, pH 7.4) three times for 5 min, blocked with 380 µL blocking buffer (3% bovine serum albumin [BSA] solution in PBS-T), and incubated further for 1 h at 37 °C. The plates were washed with washing buffer three more times for 5 min each. Serum (100 μL; standardized dilution at 1:40 in 1% BSA in PBS-T) sampled from 2 experimental groups at different time points was added to different wells and incubated in an ELISA shaker at 25 °C for 1 h. Subsequently, 100 µL of diluted mouse monoclonal antibody (MAb; 1:100 in 1% BSA in PBS-T) against glycoprotein (G) of VHSV (Enbiogene, Korea) was added to each well and kept at 4 °C overnight. To read MAbs, 100 µL of MAb (1:100) alone was added to three wells of each plate. The plates were washed three times with wash buffer on the following day and incubated with 100 µL of secondary antibody (Goat anti-mice HRP conjugate, Thermo Fisher Scientific, USA), which was freshly diluted in PBS-T at 1:2000, at 37 °C for 1 h. The plates were thoroughly washed with PBS-T five times, and substrate *O*-phenylenediamine tetra hydrochloride (OPD) solution (100 mg/mL of OPD and 40 μL of H_2_O_2_ [30% v/v] in 5 mL of citrate [50 mM]–phosphate [100 mM] buffer, pH 5) was added to each well. The plates were incubated for 20 min at 25 °C in a dark chamber. Subsequently, the reaction was stopped with 50 µL of 2 N H_2_SO_4_, and the optical density (OD) was recorded at 492 nm using a VERSA max microplate reader (Perkin Elmer, USA). Results were expressed as percentage inhibition according to the following formula: PI = 100 - (mean OD_492_ of test serum × 100)/ (mean OD_492_ of MAb).

# Competitive ELISA for quantification of specific antibody against *S. parauberis* type I

Specific antibody (anti- *S. parauberis* type I Ig) quantification in the fish sera of the experimental fish groups was done by c-ELISA according to Kole et al. (16) with necessary modifications. Briefly, 100 µL of harvested bacteria diluted in PBS (adjusted to OD_540_ = 1) was coated in triplicate in 96-well ELISA plates (Nunc, Denmark) and incubated at 4 °C overnight. Then, 50 µL of poly L-lysine (Sigma-Aldrich, USA) solution (diluted at 1:4000 in PBS) was added, and plates were incubated at 50 °C for 1 h. The plate was then washed with wash buffer (PBS-T, 0.05% tween20 in PBS, pH 7.4) three times for 5 min, blocked with 380 µL blocking buffer (3% BSA solution in PBS-T), and incubated further for 1 h at 37 °C. The plates were washed with washing buffer three more times for 5 min each. Serum (100 μL; standardized dilution at 1:40 in 1% BSA in PBS-T) sampled from two experimental groups at different time points was added to different wells and incubated in an ELISA shaker at 25 °C for 1 h. Subsequently, 100 µL of diluted (1:200 in 1% BSA in PBS-T) rabbit polyclonal antibody (PAb) against *S. parauberis* type I (Enbiogene, Korea) was added to each well and kept at 4 °C overnight. To read PAbs, 100 µL of PAb (1:200) alone was added to three wells of each plate. The plates were washed three times with wash buffer on the following day and incubated with 100 µL of secondary antibody (Goat anti-rabbit HRP conjugate, GW Vitek, Korea), which was freshly diluted in PBS-T at 1:4000, at 37 °C for 1 h. The plates were thoroughly washed with PBS-T five times, and OPD solution (100 mg/mL of OPD and 40 μL of H_2_O_2_ [30% v/v] in 5 mL of citrate [50 mM]–phosphate [100 mM] buffer, pH 5) was added to each well. The plates were incubated for 20 min at 25 °C in a dark chamber. Subsequently, the reaction was stopped with 50 µL of 2 N H_2_SO_4_, and the OD at 492 nm was recorded using a VERSA max microplate reader (Perkin Elmer, USA). The result was expressed as percentage inhibition according to the following formula: PI = 100 - (mean OD_492_ of test serum × 100)/ (mean OD_492_ of PAb).

# Competitive ELISA for quantification of specific antibody against *M. avidus*

Specific antibody (anti- *M. avidus* Ig) quantification in the fish sera of the experimental fish groups was done using c-ELISA according to Kole et al. (16) with necessary modifications. Briefly, 100 µL of *M. avidus* (4.4 × 10^4^ cells/mL) diluted in coating buffer (carbonate–bicarbonate buffer, pH 9.6) was coated in triplicate in 96-well ELISA plates (Nunc, Denmark) and incubated at 4 °C overnight. The plate was washed with wash buffer (PBS-T, 0.05% tween20 in PBS, pH 7.4) three times for 5 min, blocked with 380 µL blocking buffer (3% BSA solution in PBS-T), and incubated further for 1 h at 37 °C. The plates were washed three more times with washing buffer for 5 min each. Serum (100 μL; standardized dilution at 1:40 in 1% BSA in PBS-T) from two experimental groups at different time points was added to different wells and incubated in an ELISA shaker at 25 °C for 1 h. Subsequently, 100 µL of diluted (1:200 in 1% BSA in PBS-T) rabbit PAb against *M. avidus* YS2 strain (previously developed in our laboratory) was added to each well and kept at 4 °C overnight. To read PAbs, 100 µL of PAb (1:200) alone was added to three wells of each plate. The plates were washed three times with wash buffer on the following day and incubated with 100 µL of secondary antibody (Goat anti-rabbit HRP conjugate, GW Vitek, Korea), which was freshly diluted in PBS-T at 1:4000, at 37 °C for 1 h. The plates were thoroughly washed with PBS-T five times, and OPD solution (100 mg/mL of OPD and 40 μL of H_2_O_2_ [30% v/v] in 5 mL of citrate [50 mM]–phosphate [100 mM] buffer, pH 5) was added to each well. The plates were incubated for 20 min at 25 °C in a dark chamber. Subsequently, the reaction was stopped with 50 µL of 2 N H_2_SO_4_, and the OD at 492 nm was recorded using a VERSA max microplate reader (Perkin Elmer, USA). The result was expressed as percentage inhibition according to the following formula: PI = 100 - (mean OD_492_ of test serum × 100)/ (mean OD_492_ of PAb).

# Quantitative expression analysis of immune genes in experimental samples

# Gene-specific primers for immune-related genes were designed using Primer3Plus based on available sequences from the NCBI database and are listed in Supplementary Table 1. Olive flounder β-actin was selected as a housekeeping gene. Real-time PCR was carried out in an Exicycler^TM^ 96 Real-Time Quantitative Thermal Block (Bioneer, Korea) using SYBR Green AccuPower® PCR PreMix (Bioneer). For relative quantification of each gene, cDNA was synthesized from the head kidney, spleen, skin and gills from the experimental and naive groups and used as a template. The reaction was performed in duplicate in a final reaction volume of 20 µL with initial denaturation at 94 °C for 10 min, followed by 35 cycles of 20 s of denaturation (94 ⁰C), annealing (temperature is given in Supplementary Table 1), and scanning. The threshold cycle (Ct) value was determined using the automatic setting on the Bioneer Exicycler^TM^ 96 Real-Time PCR system. Relative quantification of immune response was estimated using the 2^- ΔΔCt^ method (25).

# Supplementary Table 1. Primers used for real-time PCR

| Target gene | GenBank acc. no | Product length | Sense primer | Antisense primer | T_a_ |
| --- | --- | --- | --- | --- | --- |
| β-actin | HQ386788 | 131 | CCTCTTCCAGCCTTCATTC | TGGTTCCTCCAGATAGCAC | 56 |
| IgM | AB052744 | 115 | GCCTCCTTCTTCTGCTCTG | CCTCAGTGGATGTTGTGATT | 56 |
| IgT | KX174302 | 150 | TAATTGTTCAGTAACTCATGCCG | GATTGAAGTGTTCCTATGCGTCT | 56 |
| pIgR | HM536144 | 478 | AAGGAGGAGGACTCTGGGTG | TGGTGATGGGTCTGGATGG | 58 |
| TLR2 | AB109393 | 100 | GCTACATCTGCGACTCTCCT | CACAGGGACACGAACAAATC | 58 |
| TLR7 | HQ845984 | 97 | CCTGGGAAATCTGGAAGAAC | TTTGAGGGAGGAGAAACTGC | 62 |
| IL-1β | AB070835 | 128 | AAAGAAGCATCACCACTGTCT | CTACTCAACAACGCCACCTT | 56 |
| IL-8 | AF216646 | 200 | TCGGCCGCTACATTAAGAGT | TTAAACGGCTTCTGACCCCATC | 58 |
| C3 | AB021653 | 233 | CTGCGCACATTCCTGAGTTA | TACTGCTGGACCATCTGCTG | 58 |
| IFN-γ | AB435093 | 126 | CTACAAGCGGCGATATGATG | GGAGGTTCTGGATGGTTTTG | 64 |
| Mx | AB110446 | 159 | TCACTGGATTTCCCAACCTC | TGTCACTCAAACTGCTGCTG | 62 |
| Caspase 3 | JQ394697 | 115 | ACATCATGACACGGGTGAAC | TCCTTCGTCAGCATTGACAC | 58 |
| Caspase 1 | KY556657 | 234 | TCAGGAGGAGGGCTGGTCTA | TGTTTCACCACCTCGTATCCC | 60 |
| IL-10 | AB685381 | 224 | ATGACTCTTCGGTCTCTCCT | TTGAAAGACTCCTCCACGCTCT | 60 |
| TNF-α | AB040449 | 113 | AAACACCTCACGTCCATCA | GCGTCCTCCTGACTCTTCT | 56 |
| CD8 | AB082957 | 108 | TAAGGGCAACACTAACACAGG | ATGAGGAGGAGGAGAAGGAG | 56 |
| T_a_: annealing temperature | | | | | |
